# Structural Basis for Chemerin Recognition and Signaling Through Its Receptors

**DOI:** 10.3390/biomedicines12112470

**Published:** 2024-10-28

**Authors:** Yezhou Liu, Aijun Liu, Richard D. Ye

**Affiliations:** 1 Kobilka Institute of Innovative Drug Discovery, School of Medicine, The Chinese University of Hong Kong, Shenzhen 518172, China; 2 Dongguan Songshan Lake Central Hospital, Dongguan Third People’s Hospital, The Affiliated Dongguan Songshan Lake Central Hospital, Guangdong Medical University, Dongguan 523326, China; 3 The Chinese University of Hong Kong, Shenzhen Futian Biomedical Innovation R&D Center, Shenzhen 518048, China

**Keywords:** chemerin, CMKLR1, GPR1, GPCR structures, adipokines

## Abstract

Chemerin is a chemotactic adipokine that participates in a multitude of physiological processes, including adipogenesis, leukocyte chemotaxis, and neuroinflammation. Chemerin exerts biological functions through binding to one or more of its G protein-coupled receptors (GPCRs), namely chemokine-like receptor 1 (CMKLR1), G protein-coupled receptor 1 (GPR1), and CC-motif receptor-like 2 (CCRL2). Of these receptors, CMKLR1 and GPR1 have been confirmed as signaling receptors of chemerin, whereas CCRL2 serves as a chemerin-binding protein without transmembrane signaling. High-resolution structures of two chemerin receptors are now available thanks to recent advancements in structure biology. This review focuses on the structural perspectives of the chemerin receptors with an emphasis on the structure–activity correlation, including key components of the two receptors for ligand recognition and conformational changes induced by chemerin and its derivative peptides for G protein activation. There are also comparisons between the two chemerin receptors and selected GPCRs with peptide ligands for better appreciation of the shared and distinct features of the chemerin receptors in ligand recognition and transmembrane signaling, and in the evolution of this subclass of GPCRs.

## 1. Chemerin and Chemerin Receptors

### 1.1. Chemerin and Its Biologically Active Peptides

**Figure 1:**
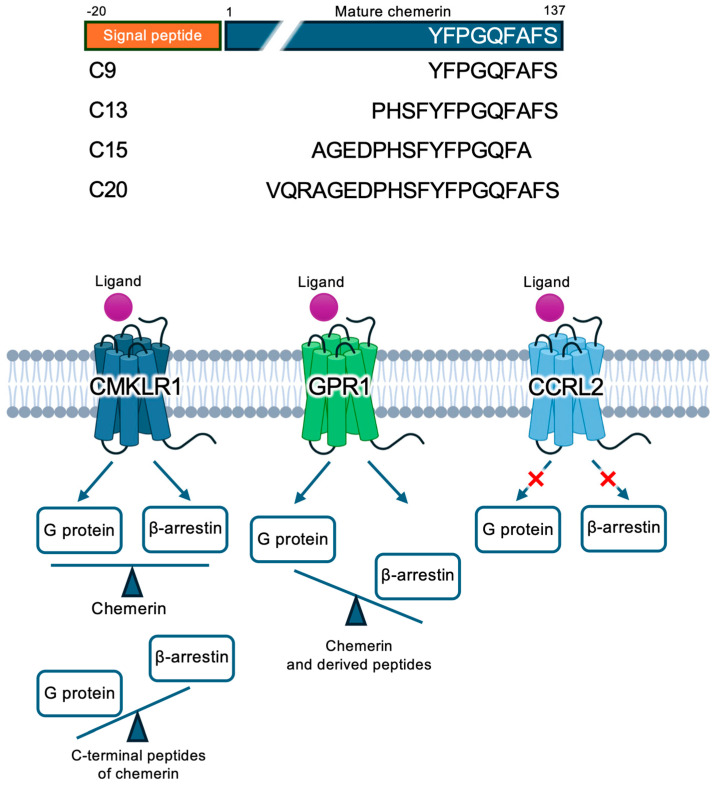
Chemerin, chemerin-derived peptides, and their receptors. Upper panel, chemerin and its derived C-terminal peptides. The N-terminal signal peptide (a.a. −21 to −1) is marked in orange. The mature form of chemerin (a.a. 1–137) is marked in teal blue. The selected C-terminal peptides of chemerin are listed. Lower panel, schematic illustration of the 3 chemerin receptors and their downstream signaling pathways. Chemerin binding to CMKLR1 elicits balanced responses of the G protein and β-arrestin signaling pathways. The C-terminal nonapeptide of chemerin (C9) induces G protein-dependent signaling through CMKLR1. GPR1 is activated by chemerin and its C9 peptide with a β-arrestin bias. Chemerin ligands bind to CCRL2 but do not induce downstream signaling.

Chemerin was initially identified as a protein product of the *retinoic acid receptor responder 2* gene, also named tazarotene-induced gene in an independent study [1,2]. It translates into a preprochemerin (163 a.a.) that undergoes proteolysis for removal of its N-terminal signal peptide (20 a.a.), resulting in a prochemerin (a.a. 21–163) with little bioactivity [3,4]. After further proteolytic removal of 6 a.a. from the C-terminus, the mature chemerin (a.a. 21–157) acquires chemotactic activity (Figure 1). Chemerin has been found to be a natural ligand of a G protein-coupled receptor (GPCR) with homology to other chemotactic receptors, and was thus named chemokine-like receptor 1, or CMKLR1 [5]. It was independently identified as an orphan receptor named ChemR23 [6,7]. Chemerin is chemotactic to immature dendritic cells, macrophages, and natural killer cells [7,8]. Cell-based and in vivo studies found proinflammatory activities of chemerin by inducing the expression of TNFα, interleukin-6, and C-reactive protein at inflammatory sites [9,10]. The expression of chemerin is associated with differentiation of adipocytes, and failure of chemerin expression has a negative impact on adipogenesis. These findings led to the assignment of chemerin as an adipokine related to obesity [11,12]. The chemerin–CMKLR1 axis has also been found to restrict microbiota-driven colonic neutrophilia and tumorigenesis through the regulation of epithelial functions [13]. Altogether, the biological activities of chemerin are increasingly appreciated.

A number of chemerin-derived peptides have been identified (Figure 1). Among these peptides, C9 (C-terminal nonapeptide of chemerin, Y^129^FPGQFAFS^137^) has shown full biological activity of mature chemerin and is often used in cell-based studies as a surrogate ligand of chemerin. C9 binds to chemokine-like receptor 1 (CMKLR1) and G protein-coupled receptor 1 (GPR1) to elicit various responses [14,15,16]. C9 has been found to induce the internalization of CMKLR1 in a clathrin-independent manner [17]. Moreover, a chemically modified C9 peptide with a longer half-life has recently been developed as a CMKLR1-targeting tracer in vivo [18]. More recent work has demonstrated that the activation of CMKLR1 by C9 peptide mitigates NLRP3 inflammasome-mediated neuronal pyroptosis in ischemic stroke [19], suggesting that C9 may have a more complicated regulatory role in inflammation. C13, the C-terminal 13-peptide of chemerin (P^125^HSFYFPGQFAFS^137^) is slightly longer than C9 to the N-terminal end and can also activate both CMKLR1 and GPR1 with slightly different potency than that of C9 (pEC_50_ of 9.05 ± 0.09 for C13, compared with pEC_50_ of 8.65 ± 0.14 for C9) [15]. C15 (A^121^GEDPHSFYFPGQFA^135^) is another synthetic peptide derived from chemerin. It lacks the last two amino acids of the C9 peptide but retains the ability to activate CMKLR1 with anti-inflammatory effects [20,21]. The C20 peptide, representing the C-terminal 20 a.a. of chemerin (V^118^QRAGEDPHSFYFPGQFAFS^137^) is an agonist of CMKLR1 with similar but less potent chemotaxis activity compared to chemerin [22]. These biologically active synthetic peptides of human chemerin exemplify the importance of the C-terminus of chemerin in receptor-mediated functions, as removal of only 2 a.a. from the C-terminal end (in C15) causes a marked change to anti-inflammatory effect.

### 1.2. Identification of CMKLR1 as a Chemerin Receptor

CMKLR1 (also termed ChemR23), initially identified as an orphan GPCR [5,6], was the first reported receptor of chemerin [2]. CMKLR1 expression was confirmed in hematopoietic tissues [5], adipocytes [11], endothelial cells [23] and vascular smooth muscle cells [24]. CMKLR1 also responds to C9, the shortest peptide from chemerin with full agonistic activity [15]. As a Class A GPCR coupled to Gi proteins, CMKLR1 activation by chemerin and derivatives leads to the inhibition of adenylyl cyclase (AC), resulting in a lower level of cyclic AMP (cAMP) inside the cells. CMKLR1 activation by chemerin and C9 also leads to calcium mobilization, phosphorylation of mitogen-activated protein kinase (MAPK), and the activation of RhoA [14,15,17,25]. Furthermore, CMKLR1 can recruit β-arrestin for receptor desensitization and internalization following activation [17,26]. Chemerin binding to CMKLR1 mediates a range of biological functions including chemotaxis of immune cells and regulation of adipocyte differentiation [2]. Specifically, the expression of CMKLR1 highlights its functions in dendritic cells (DCs) and macrophages. Chemerin, as a pro-inflammatory chemoattractant, leads to migration of DCs and macrophages through CMKLR1 in response to inflammatory cues [16].

Apart from the immune system, CMKLR1 and chemerin are highly expressed in adipose tissues. CMKLR1 activation by chemerin regulates adipogenesis, insulin signaling and adipocyte metabolism [11,27]. Given the enriched expression of chemerin and CMKLR1 in endothelial cells and vascular smooth muscle cells, a series of studies investigated the role of chemerin–CMKLR1 axis in cardiovascular system [28,29]. Published results indicate that chemerin promotes angiogenesis through CMKLR1 [23], and this has been implicated in cancer progression [19,30,31,32]. Collectively, the expression profiles and biological effects of CMKLR1 imply important roles of CMKLR1 in inflammation, obesity and cancers.

In addition to chemerin and C9, CMKLR1 can also be activated by other ligands including the chemerin C15 peptide [20], the amyloid protein Aβ_42_ [26] and the endogenous lipid mediator resolvin E1 (RvE1) [33], typically involving signaling bias (Figure 1). Of note, Aβ_42_ induces chemotaxis and β-arrestin recruitment but not calcium mobilization [26]. The C15 peptide and RvE1 are both anti-inflammatory ligands of CMKLR1 [20,33]. Delineation of the structural basis for CMKLR1-mediated physiological functions is critical to the understanding of the biased signaling mechanisms.

### 1.3. Chemerin Binding to GPR1 and CCRL2

G protein-coupled receptor 1 (GPR1) was another orphan receptor at the time of gene cloning [34] but later identified as a chemerin receptor [14,34]. GPR1 shares more than 70% of sequence homology with CMKLR1 and recognizes chemerin and its derived C9 peptide as an agonist [35,36]. C9 binds to GPR1 with a nanomolar affinity. Mature chemerin and the C13 peptide activate β-arrestin recruitment through GPR1 with a nanomolar potency [14]. As a class A GPCR, GPR1 can also activate G protein signaling events mediated by Gαi/o and Gαq/11, such as calcium mobilization and Rho/ROCK signaling; a part of these activities are sensitive to pertussis toxin (PTX) as a feature of Gi protein coupling (Figure 1). GPR1 is implicated in glucose regulation, cardiovascular disorders, and steroid hormone synthesis [4,16,36,37,38,39]. Unlike CMKLR1, GPR1 is not involved in the vasoconstriction action of chemerin [28].

C-C Chemokine Receptor-Like 2 (CCRL2) is another receptor that interacts with chemerin, albeit in a non-signaling capacity [16,40,41]. Unlike CMKLR1 and GPR1, CCRL2 does not elicit transmembrane signaling upon binding to chemerin and its peptide derivatives (Figure 1). Instead, CCRL2 acts as a scavenger receptor for chemerin with nanomolar affinity, thus regulating chemerin level in tissues. As an atypical chemokine receptor [42], CCRL2 is widely expressed in monocytes, macrophages, DCs, neutrophils, T lymphocytes, nature killer cells (NK cells), mast cells and CD34^+^ bone marrow precursor cells [41].

## 2. Structural Basis for Receptor Recognition of Chemerin and Its C9 Peptide

### 2.1. The Orthosteric Binding Pocket for Chemerin and Its C9 Peptide

**Figure 2:**
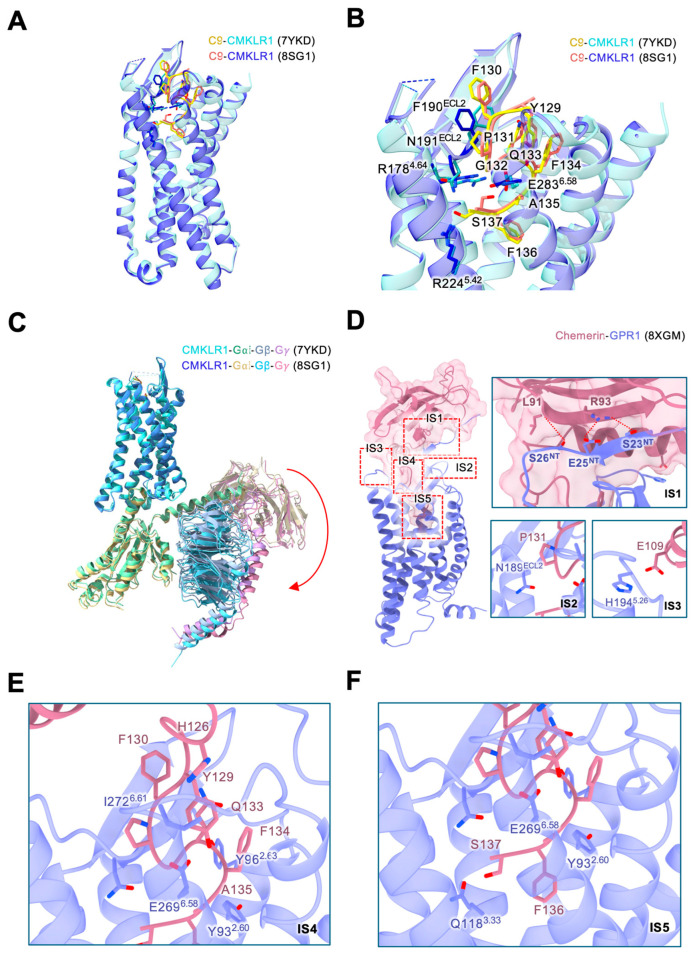
CMKLR1 recognition of chemerin and its C9 peptide. (**A**) Superimposed structures of the two solved C9-CMKLR1 complexes (PDB ID: 7YKD and 8SG1). The C9 peptide and CMKLR1 in the 7YKD structure are shown as yellow and cyan, respectively. The C9 peptide and CMKLR1 in the 8SG1 structure are shown as orange and blue, respectively. (**B**) Enlarged view of the ligand binding pocket and the interactions with the C9 peptide in the two solved structures shown above. CMKLR1 residues interacting with the ligand are marked using the Ballesteros–Weinstein numbering scheme [46]. The C9 peptide (Y129-S137) corresponds to the last 9 residues of the mature chemerin. (**C**) Comparison of the heterotrimeric Gi protein binding poses in the two C9-CMKLR1-Gi complexes. The G⍺i, Gβ, and G*γ* subunits in the 7YKD structure are colored as grass green, blue-grey and purple, respectively. The G⍺i, Gβ, and G*γ* subunits in the 8SG1 structure are colored as yellow, sky blue and pink, respectively. A red arrow indicates a downward movement of the heterotrimeric Gi proteins in the 8SG1 structure compared to the 7YKD structure. (**D**) The interaction between the full-length mature chemerin and GPR1. The full-length mature chemerin (numbered as 1-137, shown in dark pink) uses its C-terminus to insert into the transmembrane binding pocket of GPR1 (shown in purple blue). Interacting residues are shown as sticks and labeled. The 5 interaction sites (IS1-IS5) are highlighted in red boxes with dashed lines in the left panel. The panels in the right depict details of IS1 (chemerin N-terminal core interacting with GPR1 N-terminus), IS2 (chemerin interacting with ECL2 of GPR1) and IS3 (chemerin interacting with ECL3 of GPR1). (**E**) IS4 indicates chemerin Y129-A135 interacting with residues at the opening of the transmembrane pocket of GPR1. (**F**) IS5 depicts interactions of F136-S137 of chemerin with residues at the bottom of the GPR1 ligand binding pocket.

The orthosteric binding pocket of the chemerin receptor likely encompasses the transmembrane (TM) pocket and extracellular loops (ECLs). The C9 peptide has been identified as a high-affinity peptide agonist capable of inducing Gi signaling downstream of CMKLR1. With the exception of C15 which lacks the C-terminal Phe-Ser of mature chemerin, chemerin-derived C-terminal peptides exhibit strong agonistic activity at CMKLR1 and GPR1. These findings indicate that the peptides occupy the same binding pocket in the receptors as chemerin does. While the in vivo functions of these peptides remain unclear as they are not naturally cleaved chemerin products, these peptides are well-suited for structural studies. The cryo-electron microscopy (EM) structure of a C9-bound CMKLR1 in active state (complexed with Gi protein) was reported in March 2023 (PDB ID:7YKD) at an overall resolution of 2.81 Å [43]. This was followed a few months later by an independent study of the C9-bound CMKLR1-Gi structure at a resolution of 2.94 Å (PDB ID:8SG1) [44]. The two structures are highly similar (Figure 2A–C). In the transmembrane binding pocket, C9 adopts an “S” shape resembling a hook that effectively prevents dissociation from the binding site (Figure 2A,B), as confirmed by microsecond MD simulations [43]. Both structures show polar interactions between R178^4.64^, R224^5.42^, E283^6.58^ and the C9 peptide. Furthermore, the last amino acid of C9 (Ser157) falls in the hydrophilic and charged sub-pocket, while the second-to-last amino acid (F156) engages in a hydrophobic sub-pocket. Notably, residues in the ECL2, such as F190^ECL2^ and N191^ECL2^, were observed to contribute to the “balanced force” model proposed based on microsecond MD simulation (Figure 2B). However, these interactions have not been validated by EM density and mutagenesis studies [44]. There is also a downward movement of the heterotrimeric Gi proteins in the 8SG1 structure compared to the 7YKD structure (Figure 2C).

The C9–CMKLR1-Gi structure defines a TM binding pocket that can be exploited for the development of agonists to activate the Gi signaling pathway and potentially provide therapeutic benefits. However, limited structural information is available on ligand binding and activation of chemerin receptors to accurately represent the native physiological conditions. The identified TM pocket appears to be well-suited for synthetic C9 peptide but not for most endogenous ligands such as Aβ_42_, RvE1, antibodies and even mature chemerin. These ligands trigger signals beyond Gi activation, including β-arrestin recruitment, chemoattraction, and inflammatory regulation. Notably, recent structural findings revealed that chemerin recognition and binding resemble a “reverse chemokine” manner rather than peptide-like interactions [45]. This work provides further understanding of the interplay between the TM pocket and ECLs in the orthosteric pocket for signal transduction and physiological activities. Unlike chemokine receptors where the TM pocket is essential for endogenous ligand function, some endogenous ligands such as Aβ_42_ and RvE1 may not be compatible with the orthosteric membrane binding pocket, suggesting that they transmit signals through either CMKLR1 or GPR1 without occupying the TM pocket but only interact with other regions including the extracellular loops (ECLs). This specific aspect of the chemerin receptor underscores the importance of elucidating interactions between ligands and ECLs.

A notable difference between the two CMKLR1 structures is in the G protein coupling interface. Specifically, the polar interactions between the receptor and the Gβ subunit reported in one structure (8SG1) were not observed in another structure (7YKD) nor validated by functional assays. Furthermore, although both receptor structures appear to align well, there is an orientational change in the coupled Gi protein even though it is activated by exactly the same ligand, the C9 peptide (Figure 2C). The difference indicates the possibility that this region may change conformation for distinct physiological functions resulting from C9-CMKLR1 interaction, hence representing a possible mechanism for signaling bias.

### 2.2. Chemerin Interaction with Other Structural Elements of Its Receptors

With technical advancements, it is now possible to solve the structure of a chemerin receptor bound to mature chemerin. In the recently reported work [45], chemerin exhibits an atypical chemokine architecture and adopts a “reverse chemokine” two-site binding mode to engage in receptor binding. This mode of ligand-receptor interaction differs from the ones used by GPCRs with short peptide ligands such as the formyl peptide receptors (FPRs), the vasopressin receptor 2 (V2R), and the urotensin-II receptor (U2R). The interactions between mature chemerin and CMKLR1/GPR1 involve five continuous sites (IS1-IS5) that include all ECLs (Figure 2D–F, for GPR1). For IS1, the β-sheet in the globular core of chemerin accommodates the N-terminal β-strand of GPR1 through an anti-parallel hydrogen bond network formed between chemerin L91 and S26^NT^ of GPR1, chemerin R93 and E25^NT^ and chemerin R93 and S23^NT^ (Figure 2D). For IS2, chemerin P131 interacts with the ECL2 residue N189^ECL2^ (Figure 2D). IS3 is defined by a polar interaction between chemerin E109 and H194^5.26^ at ECL3 of GPR1 (Figure 2D). Deep into the receptor’s transmembrane binding pocket, the C-terminal end of chemerin is necessary and sufficient for chemerin receptor activation. Since the last two amino acids at the C-terminal end of chemerin is crucial for receptor activation (comparing the biological activities of C13 and C15), the conserved interactions between GPR1 and chemerin Y129-A135 is designated as IS4 (Figure 2E), and the interaction between GPR1 and chemerin F136-S137 is designated as IS5 (Figure 2F). Similar to the binding of C9 peptide to GPR1 and CMKLR1, multiple polar interactions involving Y93^2.60^, Y96^2.63^, E269^6.58^ and I272^6.61^ at IS4 stabilize the “S” shape binding pose of chemerin into the transmembrane binding pocket of GPR1 (Figure 2E). At IS5, the last two amino acid residues of mature chemerin form multiple polar contacts with Y93^2.60^, Q118^3.33^, and E269^6.58^ of GPR1 (Figure 2F). Collectively, the extensive interaction interface and intricate architecture facilitate the regulation of effective physiological signals. Based on this model, it is suggested that C9 represents only an artificial truncation of chemerin. Although C9 could specifically activate Gi signaling, it may not be sufficient to elicit stable and effective physiological functions.

Based on sequence and structural homology and on the shared chemotactic function, chemerin may be categorized as a novel atypical chemokine. Chemotaxis is among the most intricate cellular processes regulated in an elaborate manner. Both chemerin and chemokines act as protein ligands for chemotactic GPCRs. In contrast to small chemical molecules whose binding and dissociation are primarily controlled by chemical equilibrium through random collisions, protein ligands hold greater potential to the regulation of binding and dissociation events or even signaling events with regular frequencies of oscillation, generating a diverse spectrum of downstream signals. The structural features described above substantiate the regulation of chemotactic GPCRs beyond single activation onset to include a second layer of spatiotemporal complexity.

## 3. Structural Basis for Agonist-Induced Activation of the Chemerin Receptors

Chemerin binding to chemerin receptors relays receptor conformational change across the plasma membrane and leads to the activation of Gi proteins. Previous studies have unraveled cryo-EM structures of the C9–CMKLR1-Gi complex, C9–GPR1-Gi complex and the chemerin–GPR1-Gi complex [43,44,45]. With corroboration of receptor functional characterization and computational studies, these structural information at atomic-level provides molecular details for agonist-induced activation of chemerin receptors.

**Figure 3:**
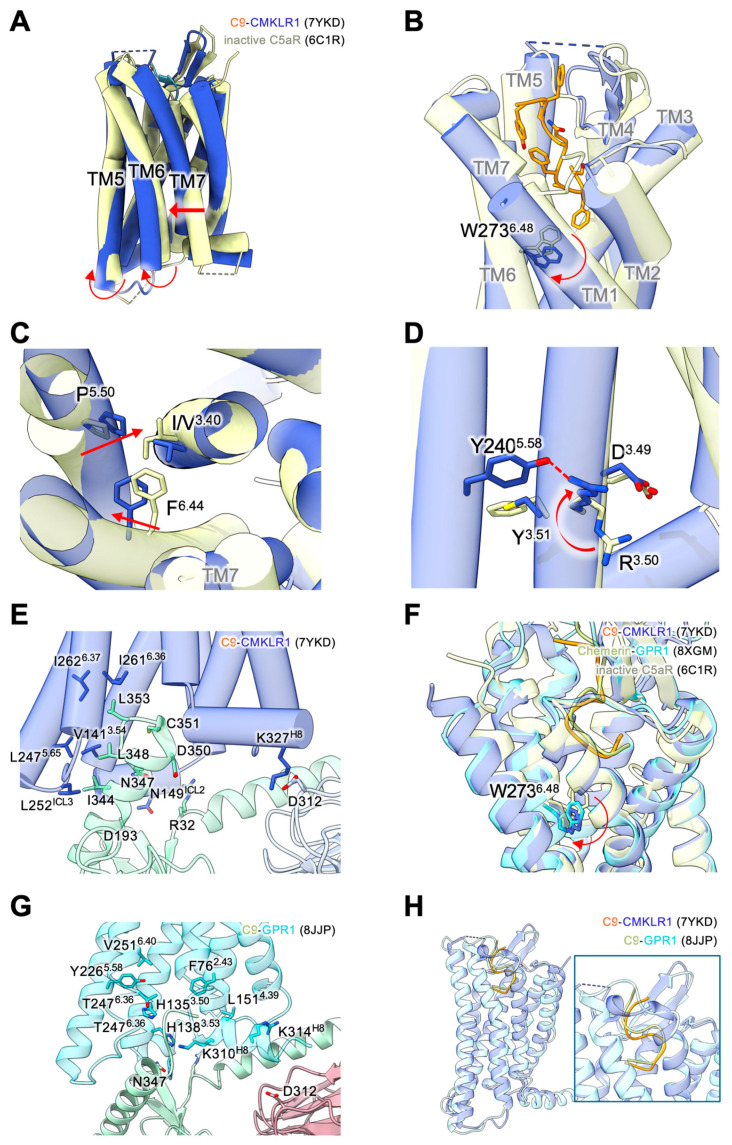
Agonist-induced activation of chemerin receptors. (**A**) Overall structural comparison between C9-bound active state CMKLR1 (PDB ID: 7YKD, blue) and inactive C5aR (PDB ID: 6C1R, light yellow) structures. Receptor conformational changes of CMKLR1 to an active state are marked with red arrows. (**B**) Upon agonist binding, the “toggle switch” W^6.48^ shows a downward orientation, further resulting in an outward movement of TM6 for an active receptor conformation. (**C**) Conformational changes at the P^5.50^-I/V^3.40^-F^6.44^ motif towards an active state GPCR conformation. Orientations of residue conformations are highlighted by red arrows. (**D**) Conformational changes at the D^3.49^-R^3.50^-Y^3.51^ motif for G protein activation. An upward rotation of R^3.50^ is highlighted by a red arrow. The red dashed line indicates the polar interaction between R^3.50^ and Y^5.58^ that secures the active receptor conformation for G protein binding. (**E**) C9-bound CMKLR1 (blue) interacting with the α subunit (teal) of the heterotrimeric Gi protein. Interacting residues are shown as sticks. (**F**) Comparison of the extent of W^6.48^ downward movement. C5aR in its inactive state is shown in pale yellow, GPR1 bound to chemerin (PDB ID: 8XGM) is shown in cyan, and CMKLR1 binding to C9 is shown in purple blue. W^6.48^ orients downwards to a greater extent in CMKLR1 than in GPR1. (**G**) C9-bound GPR1 (cyan) interacting with the α subunit (teal) of the heterotrimeric Gi protein (PDB ID: 8JJP). Interacting residues are shown as sticks. (**H**) Binding of C9 to CMKLR1 (blue) and GPR1 (cyan), respectively. The C9 bound to CMKLR1 is shown in orange, and C9 bound to GPR1 is shown in lime yellow. C9 binds deeper in the transmembrane binding pocket of CMKLR1 than in GPR1.

In the activated CMKLR1, the receptor exhibits a conformational rearrangement involving outward displacement of transmembrane helix TM6, inward movement of the intracellular end of TM7, and a distorted shift on TM5 favoring a canonical active state conformation of class A GPCR (Figure 3A). The “toggle switch” for activation, W273^6.48^ located at the bottom of the transmembrane binding pocket, shows an anticlockwise and downward rotation upon C9 binding (Figure 3B). The conformational change at this “toggle switch” ignites and relays further transmembrane conformational changes through Helix 6 towards receptor activation. Conserved structural motifs in the chemerin receptors are also identified in other Class A GPCRs and show conformational features of a GPCR in active state. Rotamer conformational changes are observed at the P232^5.50^-I/V127^3.40^-F269^6.44^ motif (Figure 3C). At the D^3.49^-R^3.50^-Y^3.51^ motif for Gαi protein coupling, an upward movement is observed for R137^3.50^, leading to the formation of a conserved hydrogen bond between R137^3.50^ and Y240^5.58^ to secure the intracellular pocket for Gαi protein (Figure 3D). For GPCR interaction, the C-terminal α5 helix of Gαi fits in the intracellular pocket of CMKLR1 formed by TM3, TM5 and TM6. Extensive polar interactions are observed between residues N347, D350, C351 and G352 of Gαi, and the side chains of S140^3.53^, N74^2.39^, R137^3.50^ and K257^6.32^ of CMKLR1, respectively. Besides, hydrophobic effects are also found involving I344, L348 and L353 of the α5 helix and V141^3.54^, L247^5.65^, L252^ICL3^, I261^6.36^ and I262^6.37^ of CMKLR1 (Figure 3E). However, the ICL3 of CMKLR1 renders less extensive interactions with Gαi. For the ICL2 of CMKLR1, N149^ICL2^ of CMKLR1 forms two hydrogen bonds with the residues R32 and D193 of αN helix of Gαi. Hence, the ICL2 is in closer proximity to the αN helix, making CMKLR1-Gαi interaction unique among class A GPCRs. Apart from Gαi interactions with the receptor, a hydrogen bond is formed between K327^H8^ of CMKLR1 and D312 of the Gβ subunit, thus strengthening the interaction between the G protein and the receptor. Together, these interacting features between Gαi and CMKLR1 are distinctive in the activation of CMKLR1.

The activation of GPR1 by the chemerin C-terminal peptide is similar to that of CMKLR1. The TM5 and TM7 of GPR1 are moved outwards, and TM6 is shifted inwards. The insertion of the C-terminus of chemerin into the transmembrane binding pocket pushes the “toggle switch” W259^6.48^ downwards to initiate a series of conformational changes for GPR1 activation (Figure 3F). Rotameric conformational changes are then induced at the P218^5.50^-I/V125^3.40^-F255^6.44^ motif. For the D^3.49^-R^3.50^-Y^3.51^ motif, it has been replaced with D134^3.49^-H135^3.50^-Y136^3.51^ in GPR1. Gαi protein binding to GPR1 involves extensive polar interactions, including α5 helix G352 and GPR1 H135^3.50^, α5 helix N347 and GPR1 H138^3.53^, αN helix R32 and GPR1 H146^ICL2^. Besides, hydrophobic effects are important in stabilizing the Gαi binding with F76^2.43^, L151^4.39^, V251^6.40^, Y226^5.58^, T247^6.36^, K310^8.49^ of GPR1 (Figure 3G). Moreover, akin to CMKLR1, a hydrogen bond between the R314^H8^ of GPR1 and D312 of the Gβ subunit is observed.

Despite the shallower insertion of the activating C-terminal peptide (C9) in GPR1 than in CMKLR1, the C9-bound GPR1 displays structural features of a GPCR in active state, providing important clues for GPR1-mediated G protein activation. The shallow binding of C9 apparently affects the signal strength of GPR1, making it less efficient for G protein signaling (Figure 3H). Specifically, the “toggle switch” W259^6.48^ in GPR1 shows downward rotation of a lesser degree than in CMKLR1 (Figure 3F). As a result, the active state conformation of GPR1 resembles an active GPCR conformational state, but with a lower amplitude in G protein activation compared to CMKLR1.

## 4. Comparison of the Chemerin Receptors and Other GPCRs in Ligand Recognition

### 4.1. Evolutionary Correlation Between CMKLR1, GPR1, and Other GPCRs

**Figure 4:**
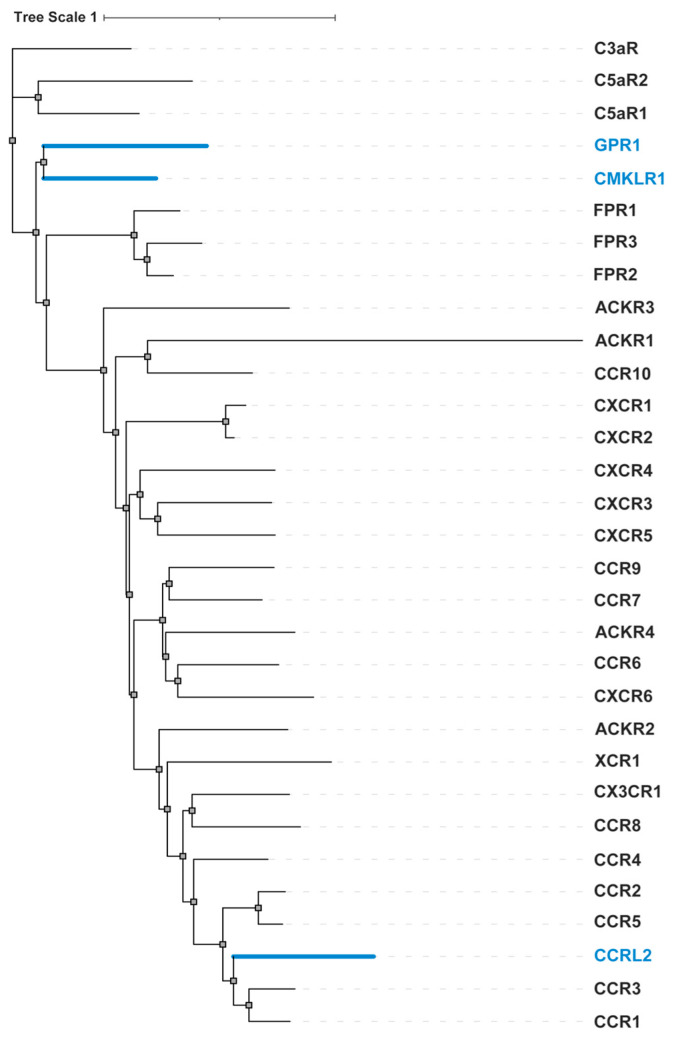
Phylogenetic analysis of the chemerin receptors, complement receptors, formyl peptide receptors, and chemokine receptors. Chemerin receptors are highlighted with blue color. The phylogenetic tree is rooted at C3aR. The graphical illustration of the tree was created using iTOL v6.9.1 [52].

The chemerin receptor family, which includes CMKLR1, GPR1, and CCRL2, is phylogenetically close to several other subfamilies of class A GPCRs with peptide or small protein ligands, including the activated complement receptor subfamily, the formyl peptide receptor subfamily, and the chemokine receptor subfamily. Among these, CMKLR1 and GPR1 are the closest homologs to complement receptors (specifically C5aR1 and C5aR2) and the formyl peptide receptors. In contrast, CCRL2 shows a closer evolutionary relationship to chemokine receptors such as CCR1 and CCR3 (Figure 4).

The complement system represents an evolutionarily ancient component of innate immunity. It has evolved into a complex network of plasma and membrane proteins that orchestrate highly organized immune responses, from pathogen recognition to leukocyte chemotaxis and activation [47]. Complement receptors, therefore, can be considered early prototypes of chemoattractant receptors that play a foundational role in regulating innate immunity [48].

Following the development of the complement system, formyl peptide receptors likely emerged as organisms developed more specialized mechanisms for combating bacterial invasion. These receptors, essential for detecting bacterial peptides, represent a critical evolutionary advancement in immune system functions such as recognition of pathogen-associated molecular patterns [49,50].

Chemerin receptors appeared next in this evolutionary sequence, with chemerin activating these receptors in a manner similar to complement-receptor interactions by using its C-terminal end. The chemerin receptor family, while retaining close ties to complement and formyl peptide receptors, also began to diversify, adapting to specialized roles in immune regulation and metabolism [16].

As the immune system became increasingly complex, the chemokine receptor family emerged, representing a sophisticated crosstalk between the innate and adaptive immune systems [51]. CCRL2, the chemerin receptor most closely related to chemokine receptors, may be an evolutionary bridge that acts as a non-functional receptor prototype during the transition from the chemerin receptor family to a fully functional chemokine receptor system.

The emergence of the chemokine receptor system marks a significant milestone in GPCR adaptation, enabling more complex immunological roles in higher organisms. This evolutionary trajectory—from complement receptors to formyl peptide, chemerin, and finally chemokine receptors—illustrates a path of increasing specialization and functional diversification. Additionally, the co-evolution of these receptors with their respective ligands and signaling pathways further highlights the dynamic nature of the evolving immune system.

### 4.2. The Chemokine Receptors

**Figure 5:**
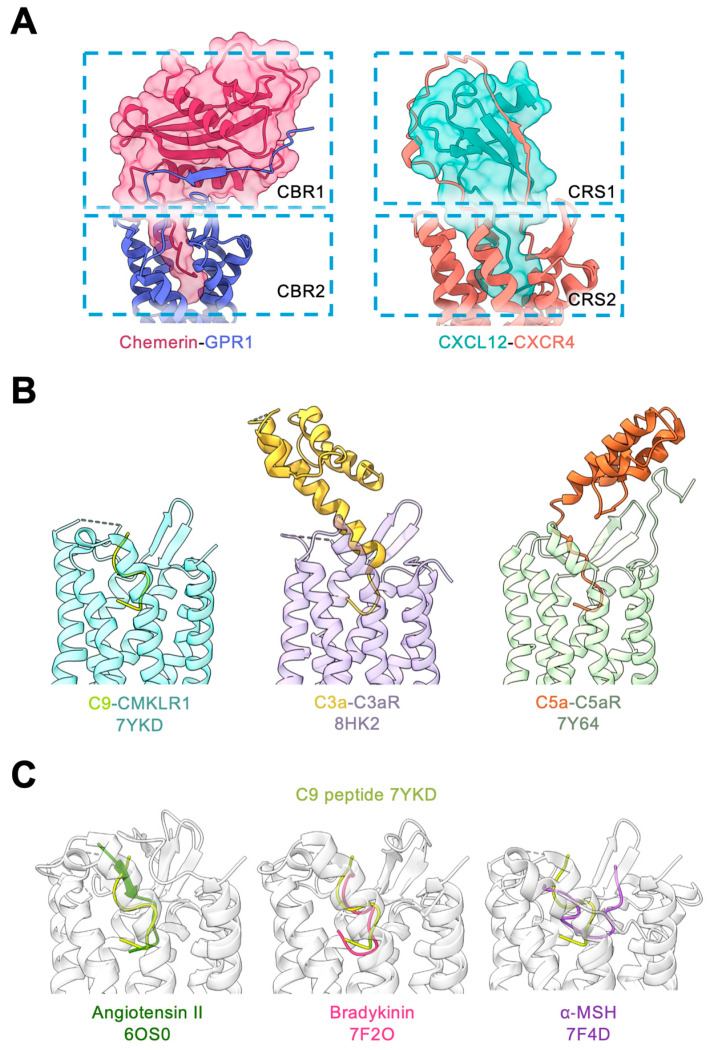
Comparison of ligand recognition of the chemerin receptors and other GPCRs. (**A**) Schematic illustration of the binding of chemerin to GPR1 (left, PDB ID: 8XGM) and chemokine CXCL12 to CXCR4 (right, PDB ID: 8K3Z, receptor N-terminus is reconstructed using AlphaFold and MD simulations). (**B**) The binding mode of C-terminus-in ligands, C9 to CMKLR1 (left, PDB ID: 7YKD), C3a to C3aR (middle, PDB ID: 8HK2) and C5a to C5aR (right, PDB ID: 7Y64), respectively. The C-terminal ends of the ligands show an “S” shape binding pose in the receptor transmembrane binding pocket. (**C**) Ligand recognition modes of peptide agonists to GPCRs. The C9 peptide bound to CMKLR1 is colored in light green (PDB ID: 7YKD) as a reference for comparison. Angiotensin II bound to AT1R is colored in dark forest green (left, PDB ID: 6OS0), bradykinin bound to the type 2 bradykinin receptor is colored in pink (middle, PDB ID: 7F2O) and α-MSH bound to melanocortin-1 receptor is colored in purple (right, PDB ID: 7F4D).

Chemotactic cytokines, or chemokines, are small proteins secreted by various cells to induce the migration of myeloid cells, predominantly leukocytes and lymphocytes [51,53]. A typical chemokine protein is stabilized by two pairs of intracellular disulfide bonds, which maintain its structure including two alpha helices, three beta strands, the connecting loops, and an exposed N-terminus that interacts with the transmembrane binding pockets of chemokine receptors (Figure 5A). Notably, chemokines and their receptors exhibit significant crosstalk in agonist recognition, with one chemokine often capable of binding to multiple chemokine receptors and vice versa. Despite this relatively low specificity, chemokine-receptor interactions follow an established model with multiple contact points [54]. The “two-site” model describes this interaction as involving two primary contact sites between the chemokine and its receptor [16,45,55,56]. The first site, known as chemokine recognition site 1 (CRS1), involves the chemokine’s globular core interacting with the receptor’s N-terminus. This initial contact is crucial for proper alignment and stabilizes the chemokine-receptor complex during activation. The second site, chemokine recognition site 2 (CRS2), entails the deeper engagement of the chemokine’s N-terminus into the receptor’s transmembrane pocket, leading to conformational changes that trigger receptor activation and subsequent intracellular GPCR signaling.

Another special type of chemokine receptor, atypical chemokine receptors, are able to recognize chemokine ligands but unable to elicit downstream signaling response [42,57]. While CMKLR1 and GPR1 can be activated by chemerin for both G protein and β-arrestin downstream responses, CCRL2 is regarded as an atypical chemokine receptor (also named ACKR5) as it binds chemokine CCL19 and chemerin without signal transduction and receptor internalization. Accumulating findings suggest that CCRL2 may cooperatively facilitate the presentation of ligands to other adjacent cells expressing CMKLR1 and GPR1 for physiological functions, finetuning the local inflammatory responses [40,58,59,60,61].

In contrast to chemokines, chemerin features a different arrangement of its intramolecular disulfide bonds. Chemerin contains two disulfide bonds, C78^chemerin^ to C97^chemerin^ and C81^chemerin^ to C115^chemerin^. Unlike chemokines, which have CC, CXC, and CX_3_C motifs, in chemerin the N-terminal Cys residues are separated by two amino acids, forming a noncanonical CX_2_C motif. The N-terminal region of chemerin forms a globular core with 2 helices and 4 anti-parallel β-sheets (Figure 2D and Figure 5A), in contrast to the C-terminal globular core structure commonly seen in chemokines. The interaction between chemerin and its receptors follows a noncanonical “two-site” binding model. The N-terminus of the chemerin receptor GPR1 is designated “chemerin binding region 1” (CBR1, corresponding to IS1-IS3, Figure 2D and Figure 5A). It engages chemerin by forming extensive beta-sheeted polar interactions with chemerin’s N-terminal globular core, thereby stabilizing the complex. Unlike chemokines that use their N-terminal region for activation of receptors via the chemokine recognition site CRS2, chemerin primarily relies on its C-terminal peptides to activate chemerin receptors through interactions with the chemerin binding region CBR2 in the receptor’s transmembrane binding pockets (corresponding to IS4 and IS5, Figure 2E,F and Figure 5A). Consequently, chemerin can be considered an atypical “reverse chemokine”.

Different from the high ligand promiscuity seen in chemokine receptors, chemerin receptors are more selective in their ligand recognition, initiating a specific set of signaling pathways and physiological functions. The unique chemerin–receptor interaction mode highlights an evolutionary divergence between the chemerin and chemokine receptor subfamilies despite their shared structural motifs and ancestral origins.

### 4.3. Receptors for C5a and C3a

C5a is a proteolytic fragment derived from the inactive complement protein C5. This anaphylatoxin, approximately 105 kDa in size, is chemotactic to phagocytes and plays a crucial role in host defense and inflammatory responses as a potent pro-inflammatory mediator [62]. C5a exerts its effects through binding to its receptors, C5aR1 and C5aR2 [63]. Similarly, C3a, another anaphylatoxin generated from the cleavage of complement protein C3, is vital for inflammation and host defense, primarily through its interaction with the C3a receptor (C3aR) [64,65]. These complement receptors, all members of the class A GPCR family, are predominantly expressed on leukocytes and are essential for mediating inflammation, chemotaxis, and host defense.

As previously discussed, C5aR1, C5aR2, and C3aR share significant sequence homology with chemerin receptors (Figure 4). The molecular mechanisms underlying complement-receptor and chemerin–receptor interactions are also similar, with both types of protein ligands utilizing their C-terminal regions to activate their respective receptors (Figure 5B). The orientations of the C-terminal regions of C3a, C5a, and chemerin within the receptor’s transmembrane pocket exhibit a similar “hook-shaped” insertion, which reaches the bottom of the transmembrane pocket [66,67]. The turn formed by the last two to four residues of the C-terminus fits snugly within the transmembrane pocket, establishing extensive polar and nonpolar interactions that drive receptor activation. Despite a ~60° variation in orientation, the C-terminal regions of these ligands share a remarkably similar interaction profile, facilitating the activation of their respective receptors.

One distinctive feature that underscores the evolutionary relationship between these receptors is the structure of their N-termini. The C3a receptor (C3aR) possesses a relatively short N-terminus, consisting of only 19 amino acids, which limits its ability to tightly wrap around the N-terminal core region of complement C3a. In contrast, the chemerin receptors CMKLR1 and GPR1 have longer N-termini of approximately 30 amino acids, enabling the formation of the chemerin binding region (CBR1) through extensive β-sheet polar interactions. C5aR1 and C5aR2, which are phylogenetically closer to chemerin receptors, also feature longer N-termini that can accommodate the binding of the helical bundles of C5a.

Collectively, these structural features provide compelling evidence for the evolutionary trajectory that positions chemerin receptors between complement receptors and chemokine receptors, reflecting their intermediary role in the evolution of GPCR-mediated immune responses.

### 4.4. Other GPCRs for Peptide Agonists

Several GPCRs bind biologically active peptides, including angiotensin II, kinins, and α-MSH. These peptide agonists utilize binding pockets that share structural similarities with those used by chemerin and its C9 peptide (Figure 5C). For instance, angiotensin II (DRVYIHPF) binds to the type 1 angiotensin II receptor (AT1R) to regulate blood pressure. Within the AT1R transmembrane pocket, angiotensin II adopts an “S”-shaped “hook” pose [68], similar to the binding configuration of the chemerin C9 peptide to chemerin receptors (Figure 5C). This “S”-shaped conformation is largely defined by the C-terminal Pro-Phe motif, which occupies space at the bottom of the receptor binding pocket and trigger receptor activation.

Similarly, kinins such as bradykinin (RPPGFSPFR) promote inflammation by activating their corresponding GPCRs, specifically the type 1 and type 2 bradykinin receptors (B1R and B2R). Bradykinin also adopts an “S”-shaped “hook” pose when binding to these receptors, with the Pro7-Phe8 motif crucially defining the turn that maximizes contact with the bottom of the receptor’s binding pocket [69]. This structural feature is shared across several GPCRs, including chemerin receptors, AT1R, and complement receptors, where a “C-terminal in” ligand posture is a common mechanism for receptor activation.

The binding pose of α-melanocyte-stimulating hormone (α-MSH) is different from the aforementioned examples of “C-terminal in” peptide ligands and lies horizontally across the extracellular surface of the melanocortin-1 receptor (MC1R) [70]. Distinct from the “C-terminal in” peptide ligands, α-MSH remains mostly planar and interacts mostly with the receptor’s extracellular loops. This binding of α-MSH into the transmembrane pocket of MC1R adapts a “U”-shape, much shallower than the “S”-shape “hook” binding pose of chemerin in chemerin receptors.

The consistent presence of the C-terminal short peptide “hook” motif across various peptide ligand-GPCR interactions provides key structural insights into receptor activation and offers a valuable framework for developing targeted peptide-based therapeutics.

## 5. Conclusions Remarks

Structural analysis of CMKLR1 and GPR1 bound to chemerin and its C-terminal C9 peptide has identified key components of agonist recognition and transmembrane signaling. These structural features place chemerin in a category similar to atypical chemokines interacting with their cognate receptors, with the exception that chemerin is biologically active in terms of G protein-coupling. With its C-terminal domain inserting into the transmembrane pocket, the binding mode of chemerin is a reversal of that of chemokines. The “two-site” model of agonist (chemokine)-receptor interaction is applicable to chemerin interaction with GPR1, and the presence of interactions between the β-sheet of chemerin and the N-terminal β-strand of the receptor GPR1, combined with the involvement of the extracellular loops, provide a mechanism that secures the activating peptide in the transmembrane binding pocket. A comparison of chemerin interaction with the chemerin receptors has led to the identification of distinct and shared features of chemokines binding to chemokine receptors, as well as C5a and C3a binding to their receptors, suggesting that chemerin and its receptors play a role in the evolution of these peptide ligands and receptors in immune response. In a recent report [71], an antibody agonist that targets CMKLR1 was characterized for its ability to induce an anti-inflammatory phenotype of macrophages, with intended therapeutic use in carcinoma progression and inflammatory bowel disease. The reported findings demonstrate the ability of a non-peptide agonist to modulate CMKLR1 conformation for selected activation of signaling pathways downstream of a chemerin receptor. In future studies, the structural features described in this review, including agonist-induced receptor conformational change leading to biased activation of the receptors, may be further explored for developing novel agonists and antagonists that target the chemerin receptors.

## Data Availability

No new data were created or analyzed in this study. Data sharing is not applicable to this article.
